# A meta-analysis of complications of thread lifting

**DOI:** 10.3389/fsurg.2026.1769458

**Published:** 2026-03-31

**Authors:** Xiaocheng Zhou, Shubo Zhuang

**Affiliations:** Department of Aesthetic Medicine, The First Affiliated Hospital of Tsinghua University, Beijing, China

**Keywords:** adverse effects, complications, cosmetic surgery, meta-analysis, non-surgical facelift, patient safety, thread lift

## Abstract

**Objective:**

This study aims to systematically review and perform a meta-analysis on the complications associated with thread lifting, a popular minimally invasive aesthetic procedure, to determine the incidence and types of adverse effects.

**Methods:**

A comprehensive literature search was conducted across major medical databases including PubMed, Embase, and Web of Science, covering all publications up to April 1, 2024. The search terms included “thread lift,” “suture lift,” “barbed suture,” “facelift,” and “nonsurgical facelift,” combined with “complications” or “adverse effects.” Only prospective or retrospective cohort studies, clinical randomized controlled trials (RCTs), and case series published in English were included. Studies were excluded if they were non-English, review articles, case reports, or conference abstracts with incomplete data. Study selection followed the Preferred Reporting Items for Systematic Reviews and Meta-Analyses (PRISMA) guidelines. Data on authors, publication year, study design, sample size, patient demographics, follow-up duration, and complications were extracted and analyzed using RevMan 5.4. Heterogeneity among studies was assessed using the I^2^ statistic.

**Results:**

Our comprehensive search initially identified 537 articles. After rigorous title and abstract screening, 213 articles were further assessed, with 26 studies ultimately included in the final analysis, representing a collective sample of 2,827 patients. The meta-analysis highlighted varying incidence rates of complications associated with thread lifts: swelling was reported in 34% of cases, visible or palpable threads in 10%, skin dimpling in 7%, and ecchymoses in 26%. The analysis also revealed high heterogeneity among the studies, with I^2^ values indicating substantial to high variability: swelling (I^2^ = 92%), skin dimpling (I^2^ = 76%), visible threads (I^2^ = 88%), and ecchymoses (I^2^ = 92%). Less common complications such as ear numbness and pinching sensation were reported in fewer studies, affecting approximately 5% and 7% of patients, respectively. Complications were further categorized into early postoperative events (occurring within the first four weeks, such as swelling, pain, and ecchymoses) and late-onset complications (persisting beyond four weeks, such as visible threads, skin dimpling, and thread migration), which may carry greater clinical significance.

**Conclusion:**

Thread lifting, while generally safe, does carry a risk of several complications, which vary widely in their occurrence. This meta-analysis provides a detailed overview of the risk profile of thread lifting procedures, highlighting the need for careful patient selection and technique mastery by practitioners. The findings underscore the importance of setting realistic patient expectations and preparing for potential adverse effects.

## Introduction

Over the last several decades, cosmetic surgery has expanded significantly due to heightened societal emphasis on aesthetic appearance (1–3). Within this burgeoning field, thread lifting has emerged as a pivotal technique, characterized by its minimal invasiveness and substantial efficacy in facial rejuvenation (4, 5). This procedure involves the strategic placement of absorbable or non-absorbable sutures beneath the skin to leverage the tension created by the threads to elevate and reposition the skin and subdermal structures, thereby delivering a pronounced tightening and lifting effect (6). The threads employed in contemporary practice vary considerably in terms of their material composition, structural design, and mechanical properties. Broadly, thread materials can be classified as permanent (e.g., polypropylene) or biodegradable [e.g., polydioxanone [PDO], polycaprolactone [PCL], and poly-L-lactic acid [PLA]], and may differ in configuration such as the presence or absence of cogs, barbs, or mesh architectures. Surgical techniques also range from simple subcutaneous insertion to more complex approaches involving dissection and fixation to deep fascia. Notable for its operational simplicity, relatively brief recovery timeframe, minimal invasiveness, and immediate visible results, thread lifting has secured its role as a cornerstone in the repertoire of modern non-surgical aesthetic interventions (7, 8).

Despite its apparent benefits, the potential for adverse effects associated with thread lifting necessitates careful consideration. The literature documents various complications, including local infections (9), migration of the threads (10), skin dimpling (11–13), tissue fibrosis (14), appearance anomalies (15), and suture breakage (16, 17). These issues not only compromise the aesthetic outcomes but may also necessitate further medical interventions, potentially imposing significant psychological and financial burdens on patients. Furthermore, the wide range of thread materials and procedural techniques introduces substantial variability in the type and frequency of complications, making a pooled analysis across studies both challenging and clinically important.

Given these considerations, our study conducts a comprehensive meta-analysis to rigorously quantify the incidence of complications associated with thread lifting. By systematically collating and analyzing data from a multitude of studies, we aim to furnish a robust scientific foundation that will aid clinicians in making informed procedural choices and enable patients to better understand the associated risks. This informed approach is intended to optimize patient outcomes and satisfaction by mitigating potential complications through enhanced procedural strategies and patient education.

## Methods

### Literature search strategy

To ensure comprehensiveness and systematic coverage, this study's literature search encompassed the following major medical databases: PubMed, Embase, and Web of Science. The search covered the period from the inception of each database until April 1, 2024. The search terms combined specific technique names and related medical terms, including “thread lift,” “suture lift,” “barbed suture,” “facelift,” “nonsurgical facelift,” as well as “complications” or “adverse effects.” To enhance the breadth of the search, both MeSH terms and free text were utilized to adapt to the search features of each database. Detailed search strategies can be found in the appendix.

### Inclusion and exclusion criteria

Inclusion criteria were set to include all prospective or retrospective cohort studies, clinical randomized controlled trials (RCTs), and case series on complications of thread lift surgery. Exclusion criteria included non-English literature, review articles, case reports, conference abstracts, and studies with incomplete data.

### Study selection

**Figure 1:**
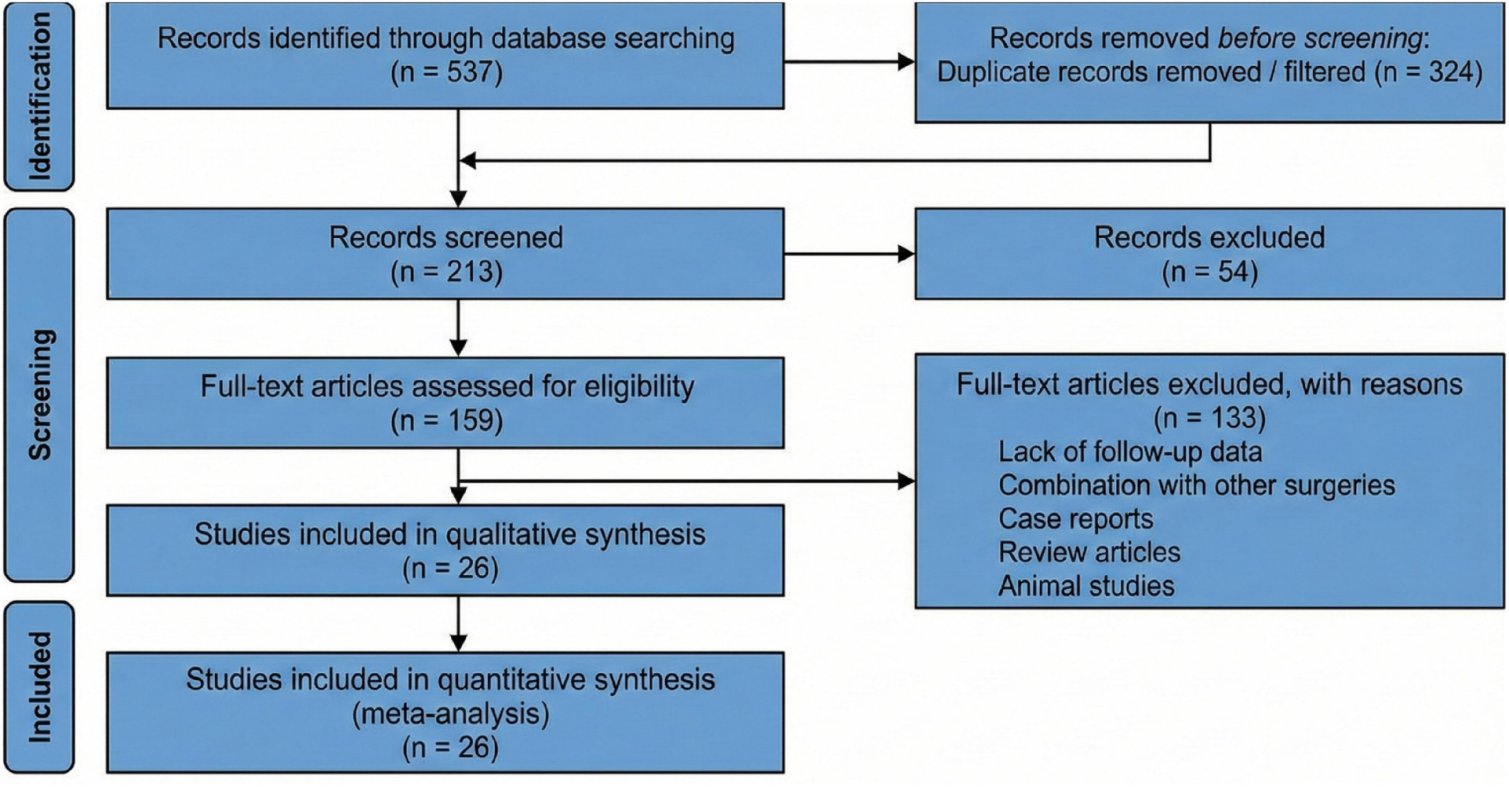
PRISMA flow diagram of study selection.

The study selection process was conducted in accordance with the Preferred Reporting Items for Systematic Reviews and Meta-Analyses (PRISMA) guidelines (18). The initial phase of the literature selection process involved a thorough screening of titles and abstracts to identify studies that potentially aligned with the specific focus of our research on thread lifting and associated complications. This step effectively eliminated any publications that did not directly address the core themes of our study. Following this initial screening, two independent researchers conducted a detailed full-text review of the remaining articles. This comprehensive evaluation was critical to accurately assess each study's relevance and adherence to our stringent inclusion and exclusion criteria. It ensured that only the most pertinent studies were considered for our analysis, thereby maintaining the integrity and scientific rigor of our research. In instances where the two reviewers disagreed on the eligibility of a particular study, a third-party expert was consulted to arbitrate and provide a decisive judgment. This arbitration process was an essential measure to guarantee impartiality and consensus in the final study selection, ensuring that all included research met our high standards for quality and relevance. The complete study selection process, including the number of records identified, screened, excluded at each stage, and ultimately included, is presented as a PRISMA flow diagram in Figure 1.

### Data extraction

For this systematic review, detailed data were meticulously extracted from each selected study to construct a robust dataset essential for our analysis. The extracted information included the names of the authors and the year the study was published, providing context and allowing for assessment of the research's recency and relevance. The type of study design was noted to evaluate the methodological quality and applicability of the findings. The sample size of each study was recorded to understand the scale and statistical power of the results. We also gathered detailed baseline characteristics of the study populations, including age and gender, which are crucial for assessing the generalizability of the findings to broader populations. The duration of follow-up was extracted to determine the longitudinal reliability of the results and to assess the long-term implications of thread lifting complications.

Furthermore, the specific types of complications reported in the studies and their respective incidence rates were carefully documented. This information is vital for understanding the risk profile associated with thread lifting procedures and for informing clinical practice. The data extraction process was carried out independently by two researchers to minimize bias and ensure data integrity. Any discrepancies encountered during the data extraction phase were resolved through direct discussion between the researchers, facilitating a consensus-driven approach to ensure the accuracy and reliability of the data used in our analysis. This meticulous process underpins the validity of our research conclusions, contributing significantly to the existing body of knowledge on cosmetic surgery complications.

### Quality assessment

The quality appraisal of the literature was based on a specific checklist, the “Quality Appraisal of Case Series Studies Checklist,” which originally consists of 20 items. In this study, 10 relevant questions from this checklist were selected for evaluation. Based on these 10 selected questions, the assessment criteria are as follows: High quality: If all 10 questions are answered affirmatively, the study is defined as high quality. Moderate quality: If eight or more of the questions are answered affirmatively, the study is defined as moderate quality. Low quality: If fewer than eight questions are answered affirmatively, the study is defined as low quality. In the event of any dispute regarding the eligibility or quality of the literature, a third party will adjudicate.

### Statistical analysis

We extracted all outcomes involved in the studies included in our research, conducting meta-analyses only on those outcomes reported in three or more articles. The meta-analyses were executed using RevMan 5.4 software, a robust tool for handling complex statistical data in systematic reviews. For assessing the incidence of complications, we used a random-effects model, which is particularly suitable given the expected variability across studies in terms of procedures, patient groups, and study settings.

To address and quantify heterogeneity, which reflects the degree to which the effect sizes vary among included studies, we employed the I^2^ statistic. This measure helps determine the suitability of pooling the individual studies’ results and interpreting the meta-analysis's overall outcome. The heterogeneity was categorized into four levels: I^2^ < 25% was considered to reflect low heterogeneity; 25% ≤ I^2^ < 50% was indicative of moderate heterogeneity; 50% ≤ I^2^ < 75% suggested substantial heterogeneity; and I^2^ ≥ 75% was associated with high heterogeneity.

Statistical significance was determined at a threshold of *P* < 0.05. This conventional cut-off provides a balance between identifying genuine effects and minimizing the risk of false-positive results. Furthermore, to investigate potential publication bias, which could skew the meta-analysis results, we utilized funnel plots. These plots are particularly effective in identifying asymmetries that could indicate bias. However, to increase the reliability of these tests, we limited funnel plot analyses to outcomes reported in ten or more studies, ensuring a robust sample size for detecting genuine trends in data dispersion. This meticulous approach to data analysis enhances the validity and credibility of our findings, providing a comprehensive understanding of the risk associated with thread lifting procedures.

## Results

### Study selection results

In the preliminary search, approximately 537 relevant articles were retrieved. After screening based on titles and abstracts, 324 articles were excluded due to irrelevance, leaving 213 articles for further evaluation. Of these, 133 articles were excluded after full-text review, primarily because they were reviews, case reports, or unrelated to the research topic. The remaining 80 articles underwent detailed full-text assessment, and ultimately, 26 studies were included in the quantitative meta-analysis. All 26 studies contributed data to at least one pooled complication outcome. The PRISMA flow diagram summarizing this process is presented in Figure 1.

### Characteristics of studies

This meta-analysis encompassed data from a total of 26 studies, comprising 2,827 patients across various investigations on thread lift procedures. The studies ranged in design, including retrospective reviews, prospective studies, and comparative analyses, conducted between 2002 and 2021. The patient cohort exhibited a wide age range, from 14 to 87 years, reflecting a diverse demographic. Follow-up durations varied from 4 weeks to 30 months, providing a comprehensive overview of complications associated with thread lift procedures across different time frames. The thread materials employed across studies included both permanent sutures (e.g., polypropylene) and biodegradable polymers (e.g., PDO, PCL, and PLA), with varying structural configurations including barbed, cogged, smooth, and mesh-type designs. See Table 1.

**Table 1:** Basic characteristics of included studies.

Author	Year	Study Design	*n*	Follow up	Mean age (range)
R. F. Abraham	2009 (19)	retrospective review	33	21 m	NA
M. S. El-Mesidy	2020 (20)	comparative prospective	20	2 m	48 (39−62)
M. Fukaya	2017 (21)	retrospective	100	12 m	45 (25–64)
S. E. Han	2016 (22)	prospective record analysis study	20	12 m	43 (32–61)
M. S. Kaminer	2008 (23)	retrospective	20	11.5 m	58 (39–73)
S. H. Kang	2017 (24)	retrospective chart review	39	6 m	45 (31–70)
H. H. Kwon	2019 (25)	retrospective chart review study	25	4 m	33–67
C. Le Louarn	2018 (26)	Retrospective clinical study	342	13.6 m	NA
H. Lee	2018 (27)	Retrospective chart review	35	12 m	NA
M. P. Ogilvie	2018 (28)	prospective review	100	6 m	62 (41–87)
Y. J. Park	2021 (29)	prospective study	73	24 m	51 (31–67)
J. D. Rachel	2010 (30)	retrospective review study	29	12 m	54 (32–68)
S. Rezaee Khiabanloo	2020 (31)	prospective chart review	58	6 m	53 (30–76)
A. Santorelli	2021 (32)	retrospective study	60	9.8 m	51 (32–71)
S. Sarigul Guduk	2018 (33)	retrospective	148	22 m	45 (21–67)
A. Savoia	2014 (34)	prospective	37	24 m	37–65
D. H. Suh	2015 (35)	retrospective chart review	31	24 w	44
M. A. Sulamanidze	2002 (36)	prospective	186	30 m	21–77
R. Wanitphakdeedecha	2021 (37)	prospective	25	12 m	30–55
K. Wattanakrai	2020 (38)	prospective	21	24 m	NA
N. Yu	2020 (39)	retrospective chart review	46	12 m	50 (38–64)
J. de Benito	2011 (40)	prospective	316	18 m	47 (28–66)
P. B. Garvey	2009 (41)	retrospective	72	24 m	57
S. Lee and N. Isse	2005 (42)	retrospective	44	9 m	NA
A. Z. E. D. Badin	2005 (43)	prospective	52	18 m	14–81
B. Lycka	2004 (44)	prospective	350	24 m	NA

m, month; y, year; w, week; NA, not available.

### Incidence of complications

To enhance interpretability, complications in this analysis were categorized according to their typical time of onset. Early postoperative events, generally occurring within the first four weeks following the procedure, included swelling, pain, and ecchymoses. These are often self-limiting and may represent expected tissue responses to thread insertion rather than true procedural complications. Late-onset complications, typically manifesting or persisting beyond four weeks, included skin dimpling or asymmetry, visible or palpable threads, thread exposure, paresthesia, and infection. These late-onset events are generally of greater clinical significance, as they may require additional intervention and have a more substantial impact on patient satisfaction.

**Figure 2:**
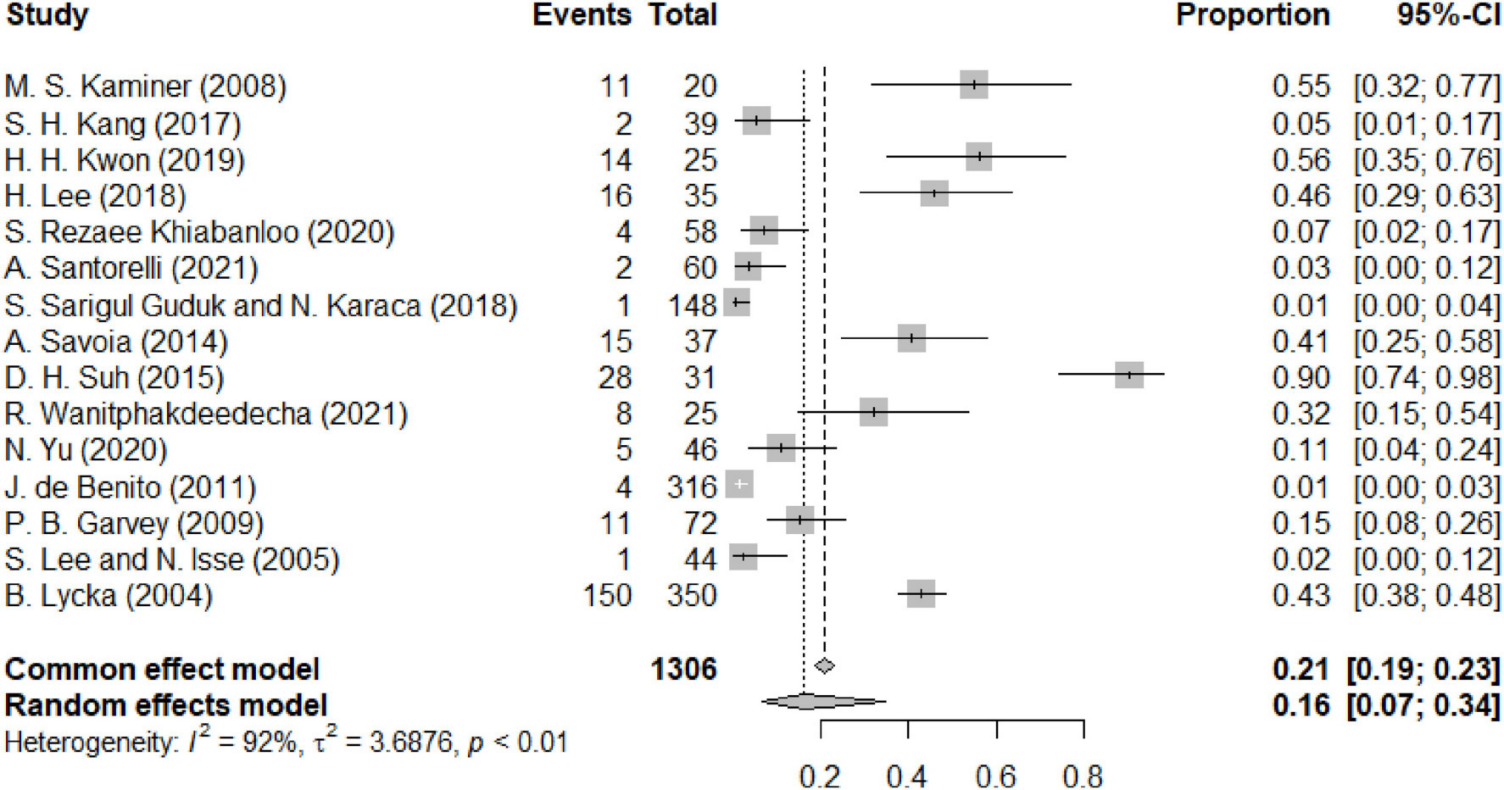
Swelling forest diagram.

Figure 2 illustrates the occurrence of swelling following thread lift procedures. Among the 26 studies included in our analysis, 15 reported swelling. Based on a random-effects model, the overall proportion of swelling following facial lift surgery ranged from 7% to 34%. However, substantial heterogeneity existed among the studies (I^2^ = 92%). As an early postoperative event, swelling typically resolved within two to four weeks across the majority of reporting studies and was largely managed with conservative measures.

**Figure 3:**
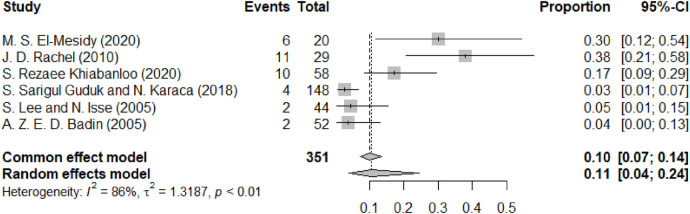
The forest diagram of pain.

 Figure 3 shows the occurrence of pain after thread lifting. A total of 6 studies reported this complication. The forest plot for the complication of pain following facial lifting shows a pooled proportion of 0.11 (95% CI: 0.04 to 0.24) using a random-effects model, with significant heterogeneity (I^2^ = 86%). The common effect model provides a slightly lower pooled proportion of 0.10 (95% CI: 0.07 to 0.14). The squares representing each study's proportion of pain events relative to their total number of cases vary in size according to the weight assigned to each study, and the diamond at the bottom indicates the overall estimated effect. Similar to swelling, pain was predominantly reported in the early postoperative period and generally subsided within the first few weeks.

**Figure 4:**
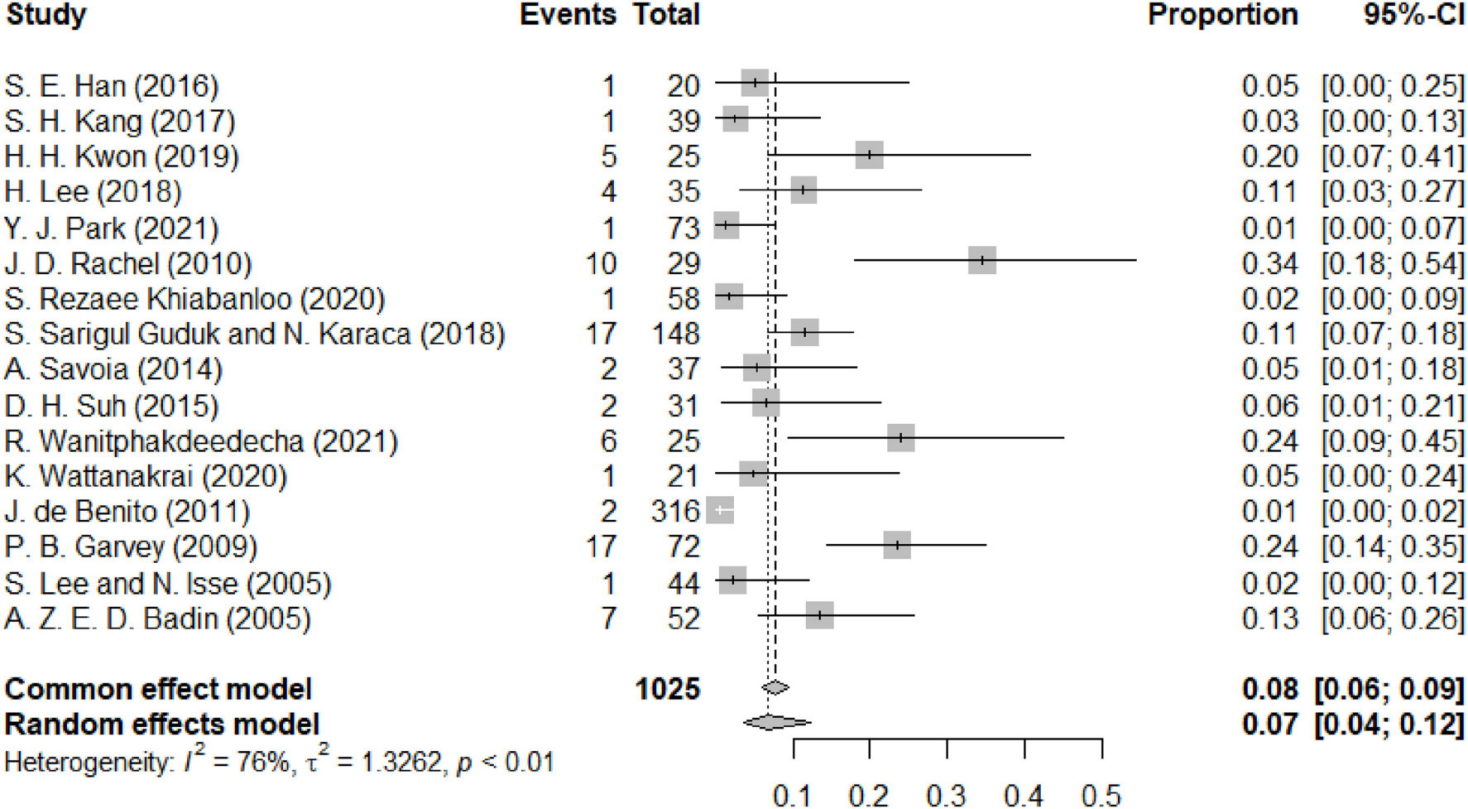
Forest diagram of skin dimpling or asymmetry pain.

 Figure 4 depicts skin dimpling or asymmetry, a complication of thread lifting that typically presents as a late-onset event. Sixteen studies reported this adverse event, mainly skin dimpling. The forest plot depicts the incidence of skin dimpling or asymmetry following facial lifting procedures across multiple studies. The random-effects model estimated a pooled proportion of 0.07 (95% CI: 0.04 to 0.12), suggesting that approximately 7% of patients experience postoperative skin dimpling or asymmetry. Heterogeneity among the studies was observed (I^2^ = 76%), indicating variability in outcomes across different research settings. Each study's effect size is represented by gray squares, with larger squares indicating greater weight in the analysis. The diamond at the bottom represents the overall pooled proportion estimate. In several studies, skin dimpling resolved spontaneously within three to six months, although a subset of patients required corrective interventions such as massage or minor revision procedures.

**Figure 5:**
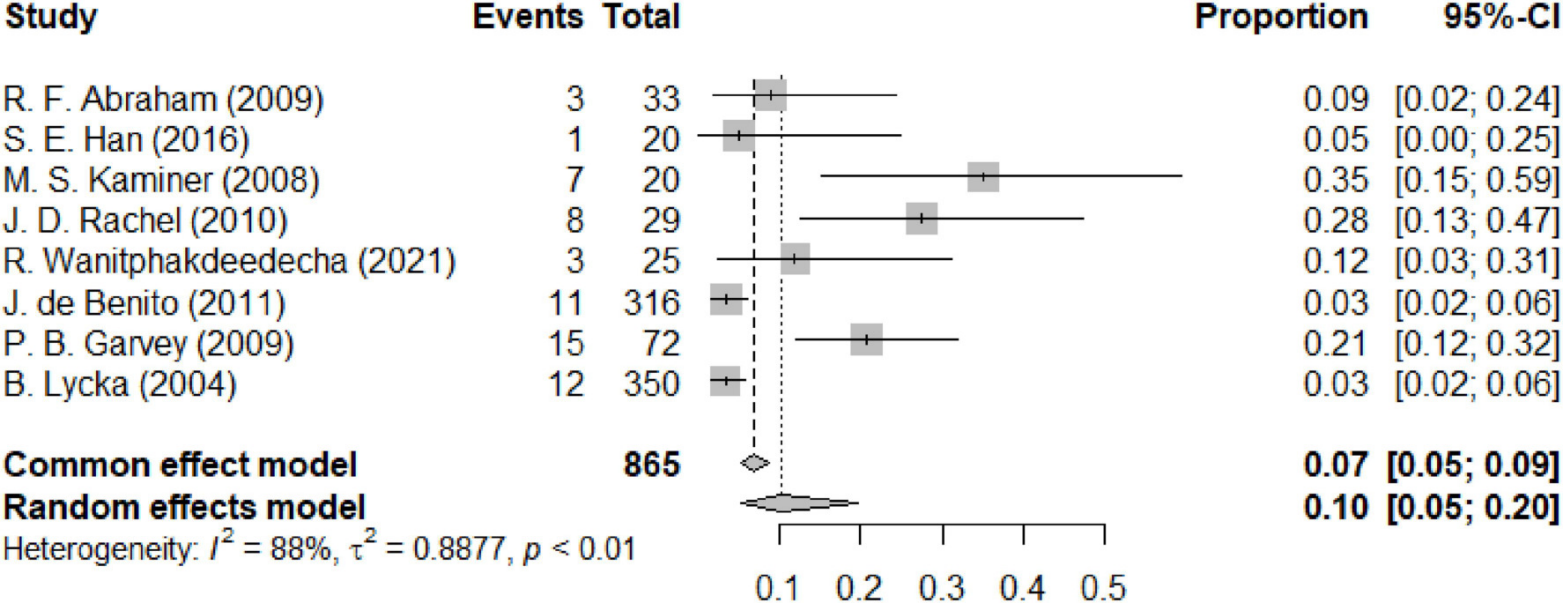
Forest map of paresthesia.

 Figure 5 is the forest plot of paresthesia. The forest plot indicates the results for the complication of paresthesia following facial lifting procedures across a selection of studies. The random-effects model estimates a pooled proportion of 0.06 (95% CI: 0.01 to 0.26), signifying a moderate average incidence rate with substantial variability, as reflected by the high heterogeneity (I^2^ = 87%). Paresthesia was reported variably as an early or late event depending on its etiology; transient numbness in the immediate postoperative period was common, whereas persistent nerve-related symptoms represented a more serious late-onset complication.

**Figure 6:**
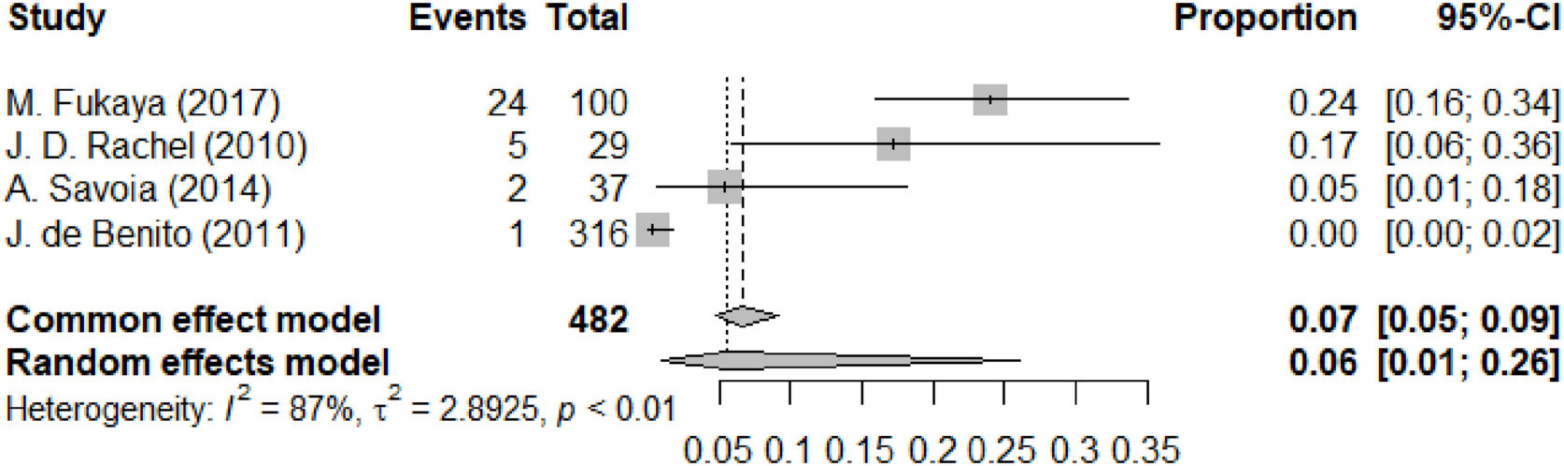
Forest diagram of visible or palpable threads/visible knots.

Figure 6 depicts another common complication. The forest plot for the complication of visible or palpable threads, also described as a visible knot, following facial lifting procedures demonstrates a random-effects model pooled proportion of 0.10 (95% CI: 0.05 to 0.20), indicating that an average of 10% of patients might experience this issue post-procedure. The common effect model suggests a slightly lower pooled proportion of 0.07 (95% CI: 0.05 to 0.09). The heterogeneity among the studies is substantial (I^2^ = 88%), suggesting considerable variation in the reported rates of this complication across different studies. The plot visualizes each study's proportional effect size with squares and their 95% CIs with horizontal lines, with the overall pooled estimate represented by the diamond at the bottom. Visible or palpable threads are classified as a late-onset complication and may be influenced by thread type, insertion depth, and the patient's skin thickness.

**Figure 7:**
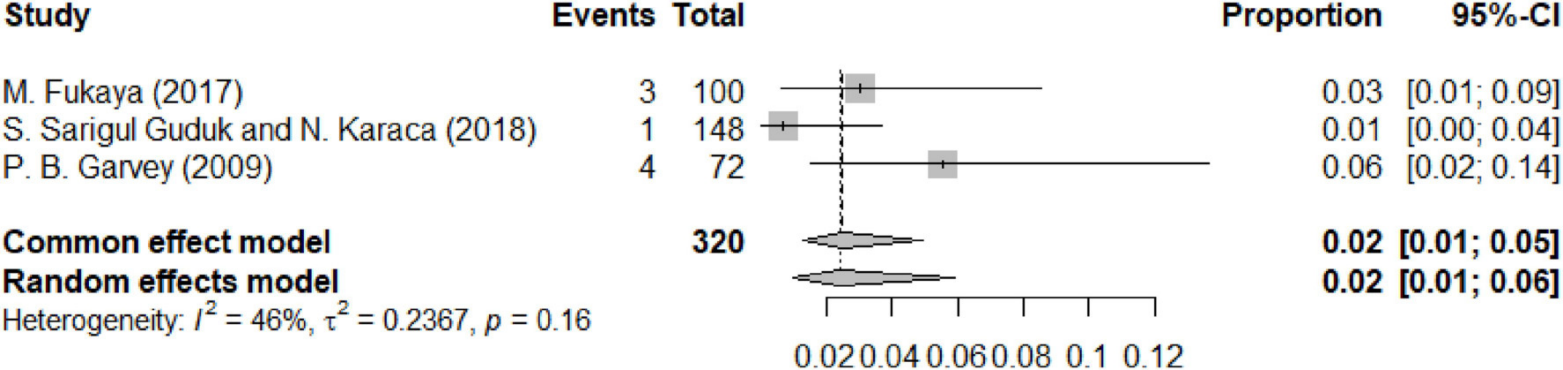
Forest diagram of infection.

Figure 7 is a meta-analysis chart of infection. Only 3 studies reported infection. The overall incidence rate was low, with a pooled effect size of 0.02 (0.01–0.06). Although infection was infrequent, it represents one of the more severe complications of thread lifting. When present, infections required antibiotic therapy and, in some cases, thread removal. More serious infectious complications such as abscess formation or deep tissue involvement, though rare, have been documented in the broader literature and underscore the importance of strict aseptic technique (45).

**Figure 8:**
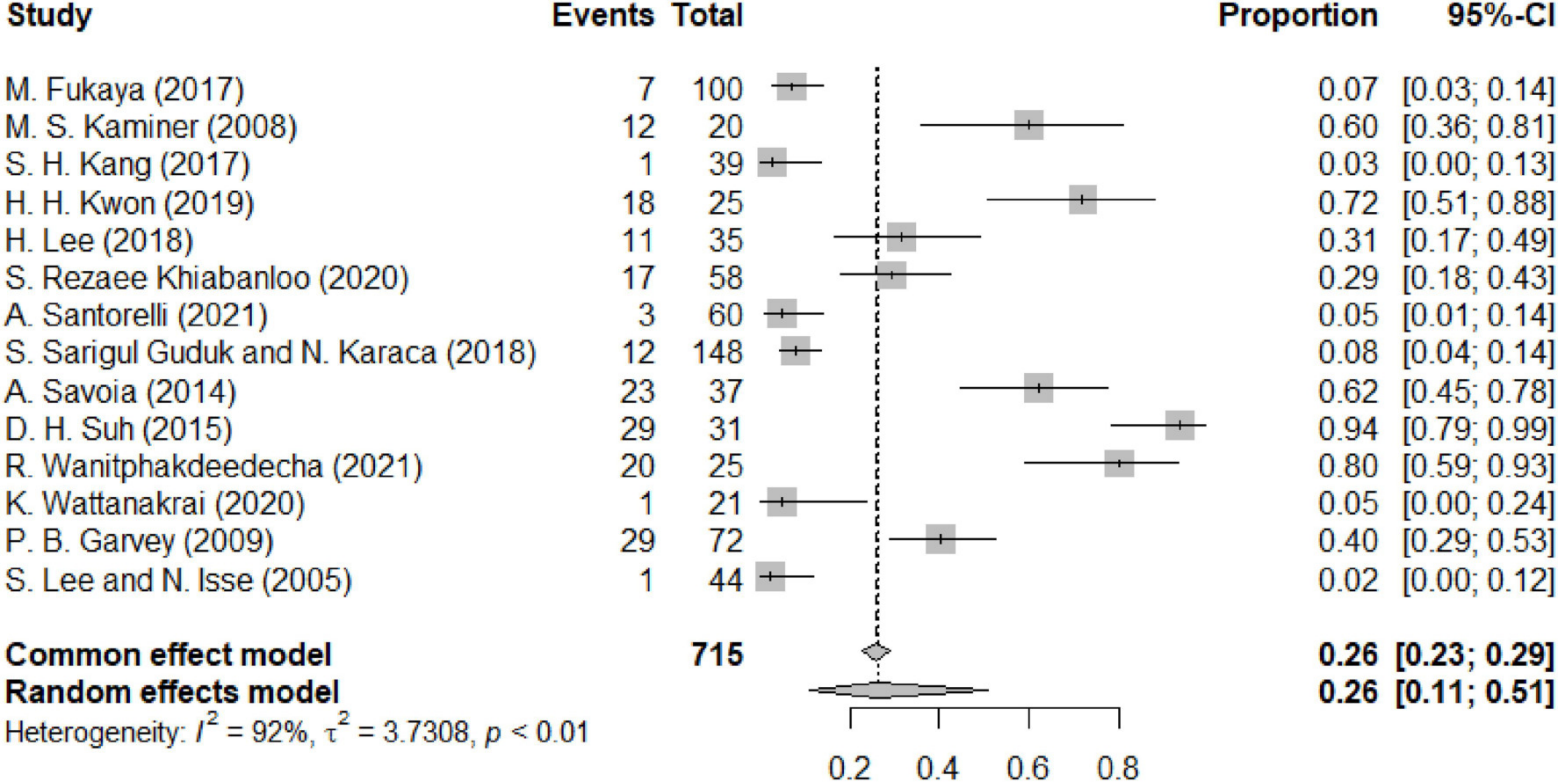
Forest diagram of ecchymoses.

Figure 8 depicts the complication of ecchymoses. The forest plot for the incidence of ecchymoses as a postoperative complication shows individual study proportions ranging broadly with a pooled estimate of 0.26 (95% CI: 0.11 to 0.51) under a random-effects model, reflecting considerable variation in outcomes, as indicated by a high I^2^ value of 92%. The common effect model gives a similar estimate of 0.26 (95% CI: 0.23 to 0.29). The plot displays each study's effect size with squares sized by weight in the analysis, and their 95% confidence intervals with horizontal lines, cumulatively represented by the diamond at the bottom for the pooled effect size. Ecchymoses, like swelling, was predominantly an early postoperative event that typically resolved within one to three weeks without requiring intervention.

**Figure 9:**
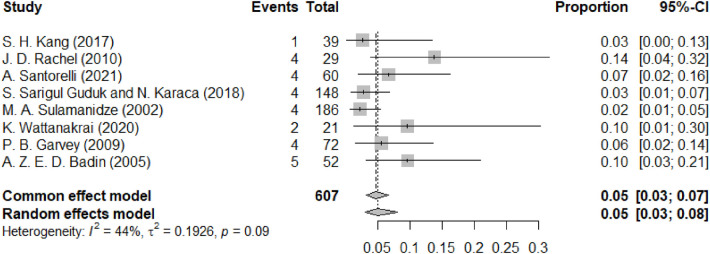
Forest diagram of thread exposure.

Figure 9 illustrates the complication of thread exposure. The forest plot summarizes the occurrence of thread exposure as a complication post-facial lifting procedures, showing an estimated pooled proportion of 0.05 (95% CI: 0.03 to 0.08) in the random-effects model and 0.05 (95% CI: 0.03 to 0.07) in the common effect model. The heterogeneity across the included studies is moderate (I^2^ = 44%), with individual study effects varying but generally indicating a low incidence of this particular complication. Thread exposure is a late-onset complication that often necessitates thread removal or repositioning and is considered moderate in severity.

Other less common complications include ear numbness, pinching sensation, malar eminence accentuation, and small hemorrhage, among others. See Table 2. Among these, certain complications such as damage to branches of the facial nerve or the parotid duct, though not reported in the included studies, have been described in isolated case reports in the literature and represent potentially severe adverse events that practitioners must remain vigilant against (46). Granuloma formation, another serious late-onset complication, has also been reported in the context of non-absorbable thread materials and warrants consideration during patient counseling (47).

**Table 2:** Other complications.

Author	Year	*n*	Complication (number of events)
M. S. Kaminer	2008 (23)	20	Ear Numbness (5)
M. S. Kaminer	2008 (23)	20	Pinching Sensation (7)
A. Santorelli	2021 (32)	60	Pinching Sensation (7)
S. H. Kang	2017 (24)	39	Malar eminence accentuation (1)
C. Le Louarn	2018 (26)	342	loss of sensitivity in part of the infra-orbital nerve distribution (2)
A. Savoia	2014 (34)	37	small hemorrhage (9)
B. Lycka	2004 (44)	350	small hemorrhage (16)
N. Yu	2020 (39)	46	Seroma (3)
P. B. Garvey	2009 (41)	72	Recurrent Laxity (9)
A. Z. E. D. Badin	2005 (43)	52	Herpes (1)
A. Z. E. D. Badin	2005 (43)	52	Inflammation of acne cyst (1)

### Publication bias assessment and literature quality assessment

**Figure 10:**
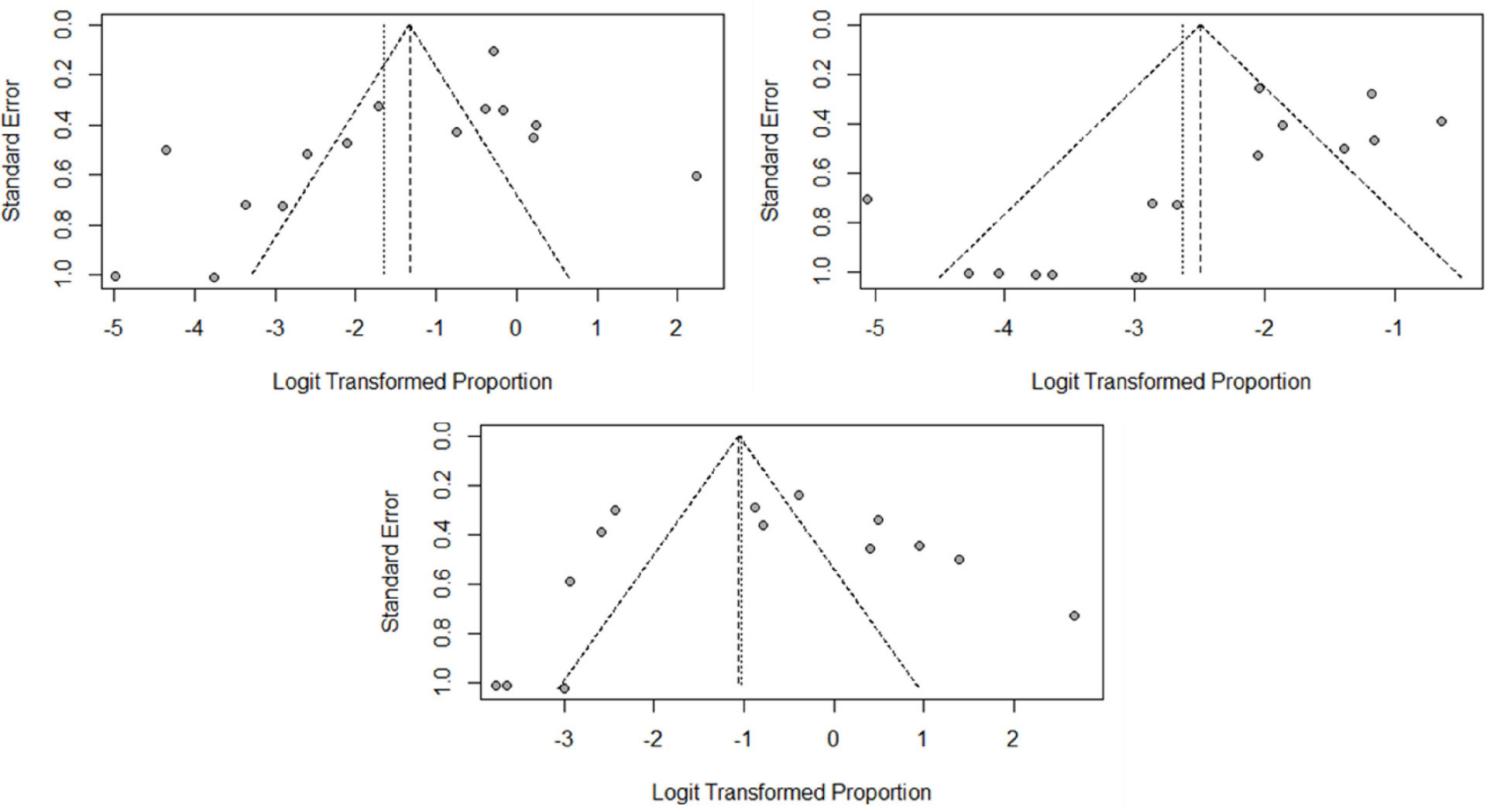
Funnel plot of swelling, skin dimpling, and ecchymoses.

Figure 10 shows the funnel plot of swelling, skin dimpling, and ecchymosis. The graph is not completely symmetrical and has a large bias. Table 3 shows the literature quality evaluation results. The overall research quality is relatively high.

**Table 3:** Literature quality evaluation.

Study	Q1	Q2	Q3	Q4	Q5	Q6	Q7	Q8	Q9	Q10	Overall Quality
R. F. Abraham (2009) (19)	l	l	l	l	l	l	l	l	l	l	low
M. S. El-Mesidy (2020) (20)	l	m	l	l	l	h	l	l	l	l	moderate
M. Fukaya (2017) (21)	l	l	l	m	h	l	m	m	l	l	high
S. E. Han (2016) (22)	l	h	l	h	l	l	h	h	m	l	high
M. S. Kaminer (2008) (23)	l	h	l	l	l	l	l	l	h	l	moderate
S. H. Kang (2017) (24)	l	l	h	l	l	l	h	l	l	m	high
H. H. Kwon (2019) (25)	l	l	l	l	l	l	l	l	l	h	moderate
C. Le Louarn (2018) (26)	l	l	l	l	l	l	l	l	l	l	low
H. Lee (2018) (27)	m	l	l	l	l	l	l	l	l	l	moderate
M. P. Ogilvie (2018) (28)	l	l	l	m	l	m	l	l	l	l	moderate
Y. J. Park (2021) (29)	h	l	l	h	l	l	l	m	l	l	high
J. D. Rachel (2010) (30)	h	l	l	l	l	l	m	m	l	l	high
S. Rezaee Khiabanloo (2020) (31)	m	l	l	l	l	l	h	h	l	l	high
A. Santorelli (2021) (32)	l	l	l	l	l	m	l	l	l	l	moderate
S. Sarigul Guduk and N. Karaca (2018) (33)	l	l	l	l	m	l	l	l	l	l	moderate
A. Savoia (2014) (34)	l	m	l	l	l	l	m	l	l	l	moderate
D. H. Suh (2015) (35)	l	l	l	h	l	h	l	l	l	l	moderate
M. A. Sulamanidze (2002) (36)	l	l	l	h	h	l	l	l	m	l	high
R. Wanitphakdeedecha (2021) (37)	l	m	l	l	l	l	l	l	h	l	moderate
K. Wattanakrai (2020) (38)	m	l	m	l	l	l	h	l	m	m	high
N. Yu (2020) (39)	m	m	l	l	l	l	l	l	m	l	high
J. de Benito (2011) (40)	m	l	l	l	l	l	m	l	l	l	moderate
P. B. Garvey (2009) (41)	l	l	h	l	l	l	m	l	l	l	moderate
S. Lee and N. Isse (2005) (42)	h	l	l	l	l	l	h	l	l	l	moderate
A. Z. E. D. Badin (2005) (43)	l	l	l	l	m	l	l	l	h	l	moderate
B. Lycka (2004) (44)	l	l	l	l	m	l	h	l	l	l	moderate

L, low; m, moderate; h, high.

Q1–Q10:

1. Is the objective of the study clearly defined?
2. Are the case selection criteria clearly described and justified?
3. Does the study include a consecutive or complete series of cases, or is there an explanation for any cases omitted?
4. Was the data collected prospectively, or was there a clear plan for data analysis articulated before the study?
5. Were data collection methods consistent and standardized across all cases?
6. Were the outcomes assessed using objective criteria or measurement tools?
7. Were all pre-specified outcomes reported on?
8. Was the follow-up duration sufficient to observe the outcomes of interest?
9. Was the dropout rate reported, and were dropouts analyzed?
10. Were potential confounding factors considered and controlled for in the study?

## Discussion

The impetus for conducting this study stemmed from a growing interest in the aesthetic community regarding the safety and efficacy of thread lifting, a procedure that has gained rapid popularity due to its minimally invasive nature and immediate results. This meta-analysis was initiated to systematically assess the range and frequency of complications associated with thread lifting, a subject of increasing concern amongst both practitioners and patients. Our findings have elucidated that while thread lifting is generally a safe procedure, complications such as swelling, skin dimpling, visible threads, and ecchymoses occur at variable rates. Notably, the incidence rate for swelling was found to be the highest, reported at 34%, followed by ecchymoses at 26%. These results underscore the necessity for thorough pre-procedural planning and patient counseling to mitigate these risks effectively.

An important consideration in interpreting these pooled complication rates is the distinction between early postoperative events and late-onset complications. Swelling (34%), ecchymoses (26%), and pain (11%) are typically self-limiting phenomena that occur within the first four weeks and resolve with conservative management. These outcomes, while clinically relevant, may represent expected tissue responses to the mechanical trauma of thread insertion rather than true procedural complications (45). In contrast, complications such as visible or palpable threads (10%), skin dimpling (7%), paresthesia (6%), and thread exposure (5%) tend to present or persist beyond the early postoperative period. These late-onset events carry greater clinical significance because they may necessitate corrective procedures, cause sustained patient dissatisfaction, and are more reflective of technique-related or material-related factors. This temporal distinction is critical for practitioners when counseling patients, as it allows for more nuanced discussions regarding expected postoperative course vs. complications warranting clinical concern.

Regarding the severity of complications, the adverse events identified in this meta-analysis can be broadly stratified into mild, moderate, and severe categories. Mild complications include swelling, ecchymoses, and transient pain, which are self-resolving and require minimal or no intervention. Moderate complications encompass skin dimpling, visible threads, and thread exposure, which may persist for weeks to months and occasionally require corrective procedures such as massage, thread repositioning, or removal. Severe complications, though rare in our pooled analysis, include deep infection (pooled incidence 2%) and persistent paresthesia suggestive of nerve injury. Although not reported in the 26 studies included in our quantitative synthesis, the broader literature has documented serious adverse events including damage to branches of the facial nerve, parotid duct injury, and granuloma formation, particularly in association with non-absorbable thread materials (46, 47). These severe complications, while infrequent, underscore the importance of thorough anatomical knowledge and meticulous technique.

Our study corroborates several findings from previous research, primarily confirming that thread lifting, while effective, is not devoid of risks (18, 48, 49). Similar studies in the past have highlighted complications like minor swelling (40, 44) and bruising (35, 41) as common immediate postoperative occurrences, which aligns with our observations of high incidence rates for these issues. Additionally, the complications related to thread visibility and migration reported in earlier studies were also consistently observed in our analysis, further validating the persistent nature of these complications across different study cohorts and procedural techniques.

It is also worth noting that this meta-analysis did not conduct a subgroup analysis based on thread material type (e.g., PDO, PCL, PLA, or polypropylene) or procedural technique (e.g., simple insertion vs. fascial fixation). The included studies employed a wide variety of thread types and surgical approaches, and the majority did not provide material-specific complication data amenable to separate pooling. This heterogeneity in thread materials and techniques is likely a major contributor to the high I^2^ values observed across most outcomes. Prior research has suggested that biodegradable materials such as PDO may be associated with different complication profiles compared to permanent sutures such as polypropylene, and that thread configuration (barbed vs. smooth, cogged vs. mesh) may also influence complication rates (50). Similarly, the duration and magnitude of the lifting effect may vary substantially across thread materials, which can in turn affect patient satisfaction. Although a dedicated analysis of patient satisfaction was beyond the scope of the present study, existing evidence suggests that satisfaction is closely linked to both the longevity of results and the occurrence of adverse events (51). Future meta-analyses with access to more granular, material-specific data would be well-positioned to address these important clinical questions.

Despite its strengths, our study has several limitations that warrant mention. First, the inherent heterogeneity of the included studies, in terms of both procedural techniques and patient demographics, may have influenced the complication rates observed. The variability in thread types used, practitioner skill, and patient skin types across studies could have contributed to the differences in complication incidences. Second, many outcomes classified as “complications” in this analysis, particularly swelling, ecchymoses, and pain, may overlap with expected postoperative sequelae depending on their timing and severity. Because the included studies varied in follow-up duration and in how they defined and recorded these outcomes, the pooled incidence rates for early-onset events should be interpreted with caution, as they may overestimate the frequency of clinically meaningful complications. Moreover, our analysis was confined to studies published in English, potentially omitting relevant data from non-English publications that could provide additional insights into the complications associated with thread lifting. The absence of subgroup analyses stratified by thread material, thread configuration, or surgical technique represents a further limitation, as these variables are known to influence complication profiles. Finally, the retrospective nature of many included studies may introduce recall bias, particularly in self-reported outcomes such as pain or minor complications, which might not always be accurately documented.

Given the findings and the limitations noted, it is imperative for practitioners to approach thread lifting with a critical eye towards optimizing techniques and selecting candidates. Practitioners should be well-versed in the various suture materials and techniques to tailor their approach to the individual patient's anatomy and desired outcomes, potentially reducing the incidence of complications. Patient selection should also involve a thorough assessment of skin quality and a detailed discussion about realistic expectations and possible adverse outcomes. Further research, particularly prospective studies with standardized protocols, material-specific reporting, assessment of patient satisfaction outcomes, and longer follow-up periods, will be crucial in advancing our understanding of the long-term safety and efficacy of thread lifting.

## Conclusion

This meta-analysis has highlighted the complications associated with thread lifting, underscoring its effectiveness as a minimally invasive aesthetic procedure alongside its potential risks. Swelling, ecchymoses, visible threads, and skin dimpling emerged as common complications, with important distinctions between early self-limiting events and late-onset complications of greater clinical significance. Our findings stress the importance of thorough pre-procedural consultations and realistic discussions with patients about the risks involved, including the expected postoperative course and the possibility of complications requiring further intervention. By improving practitioner skills, refining thread material selection, and enhancing patient understanding, the safety and satisfaction outcomes of thread lifting can be improved. Future research should focus on standardizing methods, reporting material-specific outcomes, incorporating patient satisfaction measures, and broadening patient demographics to further understand and refine the procedure's safety profile.

## Data Availability

The original contributions presented in the study are included in the article/Supplementary Material, further inquiries can be directed to the corresponding author.
